# Brain [^18^F]FDG uptake patterns in type 2 diabetes: new phenotypes relating to biomarkers of cognitive impairment

**DOI:** 10.1093/braincomms/fcaf213

**Published:** 2025-06-12

**Authors:** Queralt Martín-Saladich, Deborah Pareto, Rafael Simó, Andreea Ciudin, Carolina Aparicio, Khadija Hammawa, Elena de la Calle Vargas, Santiago Aguadé-Bruix, Marina Giralt, Clara Ramirez-Serra, Miguel A González Ballester, José Raul Herance

**Affiliations:** Medical Molecular Imaging Research Group, Vall Hebron Research Institute (VHIR), Nuclear Medicine and Radiology Departments, Vall d'Hebron University Hospital (VHUH), Autonomous University Barcelona (UAB), Barcelona 08035, Spain; Department of Information and Communication Technologies, Pompeu Fabra University, 08018 Barcelona, Spain; Department of Neuroradiology Section, Radiology, IDI, VHUH, 08035 Barcelona, Spain; Neuroradiology Group, VHIR, 08035 Barcelona, Spain; Diabetes and Metabolism Research Group, VHIR, 08035 Barcelona, Spain; Department of Endocrinology, VHUH, UAB, 08035 Barcelona, Spain; Centro de Investigación Biomédica en Red de Diabetes y Enfermedades Metabólicas Asociadas (CIBER-DEM), Instituto de Salud Carlos III, 28029 Madrid, Spain; Diabetes and Metabolism Research Group, VHIR, 08035 Barcelona, Spain; Department of Endocrinology, VHUH, UAB, 08035 Barcelona, Spain; Centro de Investigación Biomédica en Red de Diabetes y Enfermedades Metabólicas Asociadas (CIBER-DEM), Instituto de Salud Carlos III, 28029 Madrid, Spain; Medical Molecular Imaging Research Group, Vall Hebron Research Institute (VHIR), Nuclear Medicine and Radiology Departments, Vall d'Hebron University Hospital (VHUH), Autonomous University Barcelona (UAB), Barcelona 08035, Spain; Medical Molecular Imaging Research Group, Vall Hebron Research Institute (VHIR), Nuclear Medicine and Radiology Departments, Vall d'Hebron University Hospital (VHUH), Autonomous University Barcelona (UAB), Barcelona 08035, Spain; Department of Information and Communication Technologies, Pompeu Fabra University, 08018 Barcelona, Spain; Medical Molecular Imaging Research Group, Vall Hebron Research Institute (VHIR), Nuclear Medicine and Radiology Departments, Vall d'Hebron University Hospital (VHUH), Autonomous University Barcelona (UAB), Barcelona 08035, Spain; Medical Molecular Imaging Research Group, Vall Hebron Research Institute (VHIR), Nuclear Medicine and Radiology Departments, Vall d'Hebron University Hospital (VHUH), Autonomous University Barcelona (UAB), Barcelona 08035, Spain; Clinical Biochemistry Research Group, VHIR, 08035 Barcelona, Spain; Biochemical Core Facilities, VHUH, UAB, 08035 Barcelona, Spain; Clinical Biochemistry Research Group, VHIR, 08035 Barcelona, Spain; Biochemical Core Facilities, VHUH, UAB, 08035 Barcelona, Spain; Department of Information and Communication Technologies, Pompeu Fabra University, 08018 Barcelona, Spain; Catalan Institution for Research and Advanced Studies ICREA, 08010 Barcelona, Spain; Medical Molecular Imaging Research Group, Vall Hebron Research Institute (VHIR), Nuclear Medicine and Radiology Departments, Vall d'Hebron University Hospital (VHUH), Autonomous University Barcelona (UAB), Barcelona 08035, Spain; Centro de Investigación Biomédica en Red de Bioingeniería, Biomateriales y Nanotecnología (CIBER-BBN), Instituto de Salud Carlos III, 28029 Madrid, Spain

**Keywords:** insulin resistance, brain function, cognitive impairment, hyperinsulinaemic euglycaemic clamp, [^18^F]FDG-PET imaging

## Abstract

Previous studies in patients without Type 2 diabetes suggest that brain hypo- and hypermetabolic regions may indicate risk for cognitive disorders. We aimed to study these brain glucose uptake patterns in Type 2 diabetes to assess cognitive disorder risk and improve personalized management. Six hyper- and three hypometabolic regions were obtained through statistical parametric mapping, with cerebellar vermis and right superior temporal gyrus being the most relevant areas, respectively. Such allowed identification of two phenotypes via *k*-means clustering: brain hypometabolic dominant (bU[−]) and hypermetabolic dominant (bU[+]). bU[−] displayed elevated markers of both Type 2 diabetes and cognitive disorders, specifically of secreted frizzled-related protein 1, a protein related to different neuronal pathologies. A classifier was developed (area under the curve = 0.84, true positive rate = 0.81 and true negative rate = 0.78) using a combination of biochemical features. Type 2 diabetes patients exhibit hypo- and hypermetabolic brain regions that phenotype into bU[−] and bU[+] by using the relationship between right superior temporal gyrus and cerebellar vermis, which defines the transition from one phenotype to the other. We suggest bU[−] patients are exposed to a higher risk of developing cognitive disorders based on the alteration of secreted frizzled-related protein 1 due to progressed type 2 diabetes, which can be identified using the proposed biomarker-based classification model.

## Teaser

Martín-Saladich *et al.* studied brain glucose hypo- and hypermetabolism in Type 2 diabetes, linking insulin resistance to risk of cognitive impairment. Brain glucose uptake patterns classified patients as insulin sensitive or resistant, with the latter being associated with markers of cognitive disease. A biomarker-based, non-invasive model enabled at-risk patient detection.

## Graphical Abstract

**Graphical Abstract:**
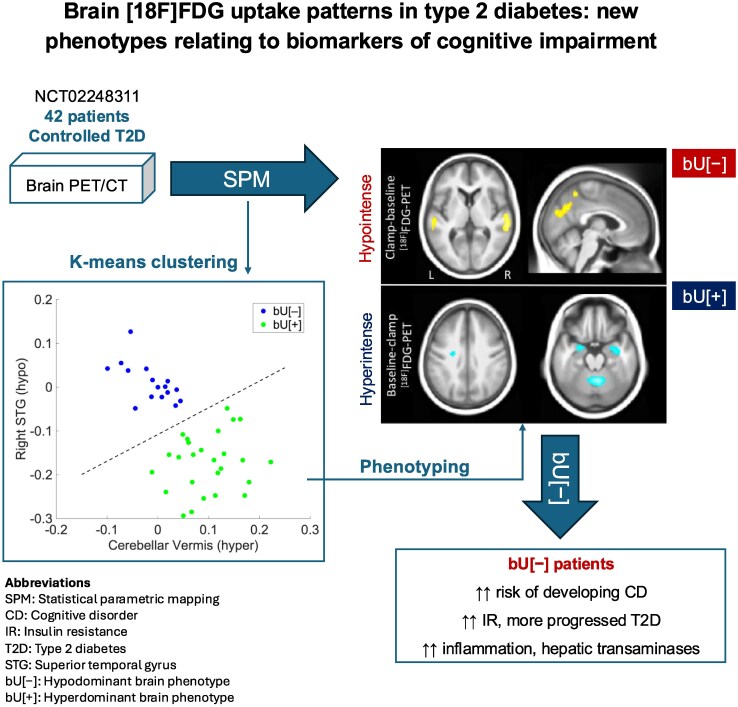

## Introduction

The brain is a complex organ with high energetic demands, accounting for ∼20% of the whole-body fuel budget.^1^ The main metabolic pathway for energy consumption is glucose oxidation, which is insulin independent and is used mostly for synaptic processes between neurons and astrocytes.^2^ Appropriate interactions between neuron and astrocytes guarantee proper optimal motor, sensory and intellectual function,^3^ whereas impaired glucose metabolism in the brain can result in the development of cognitive disorders (CDs) such as Alzheimer’s disease.^4^ Thus, brain glucose uptake patterns observed on ^18^F-fluorodeoxyglucose positron emission tomography ([^18^F]FDG-PET) images have been widely used as an *in vivo* biomarker of neurodegeneration in several CDs, such as Parkinson’s disease and Alzheimer’s disease.^5,6^ For example, some Alzheimer’s disease patterns include hypometabolism in the inferior parietal lobe, posterior cingulate cortex and medial temporal lobe,^7^ and hypermetabolism in the cerebellum and primary olfactory cortex.^8^ Additionally, brain hypermetabolism has been considered a potential compensatory reaction against initial brain damage, a transient phenomenon in disease progression in patients with obesity tightly related to Type 2 diabetes.^9^ Furthermore, glucose hypermetabolism can be produced by diverse factors unrelated to CD, making it crucial for the interpretation of the disease based on the patient’s clinical history.^10^

Under Type 2 diabetes conditions, plasma glucose cannot be properly uptaken into neurons and astrocytes due to insulin resistance (IR)^11^ which causes a decline in their synaptic processes and affects cognitive function.^12-14^ Consequently, Type 2 diabetes is considered a risk factor for CD including Alzheimer’s disease, with some authors considering it as Type 3 diabetes, due to glucose dysregulation and impaired insulin signalling.^15-18^ However, some other authors conclude that although Type 2 diabetes is associated with Alzheimer’s disease in terms of brain glucose biodistribution, there are subgroups of patients with Type 2 diabetes with brain pathologies besides Alzheimer’s disease, such as psychiatric disorders, neurodegeneration or encephalopathy.^19,20^ Therefore, different phenotypes of patients with Type 2 diabetes in terms of CD coexist, suggesting that diabetes-related dementia may be considered a distinct entity from Alzheimer’s disease itself. Thus, we hypothesized that different phenotypes of patients with Type 2 diabetes can be determined in terms of insulin-mediated glucose uptake patterns in brain regions by using [^18^F]FDG-PET imaging in combination with a hyperinsulinaemic euglycaemic clamp (HEC). Using standard [^18^F]FDG-PET conditions, patients with Type 2 diabetes display different glucose uptake patterns in insulin-sensitive (IS) organs, such as the heart and the brain due to IR. Thus, some brain areas exhibit decreased or increased [^18^F]FDG uptake, related to IR or IS areas, respectively, due to impaired insulin-mediated glucose metabolism. The areas with higher or lower [^18^F]FDG uptake are considered hyper- or hypometabolic, respectively.

As a result, we propose that brain phenotypes that might relate to altered neurodegenerative biomarkers could be identified with [^18^F]FDG-PET imaging. Furthermore, the link between brain disorders and abnormal glucose metabolism has been studied,^21^ suggesting that glucose hypo- and hypermetabolism in cerebral regions correlate with cognitive impairment.^7,8,22^ Thus, the aim of the present study was (i) to determine whether different hypo- and/or hypermetabolic glucose uptake patterns can be identified in the brain for the first time in patients with Type 2 diabetes, (ii) to phenotype patients with Type 2 diabetes according to the glucose metabolism in brain regions for the first time and evaluate their risk exposure due to abnormal CD biomarkers and (iii) to build a classification model to predict brain phenotypes based on accessible biochemical data.

## Materials and methods

### Patient characteristics

Fifty-one subjects with Type 2 diabetes who met the inclusion criteria were asked for participation in the clinical trial and, thus, were recruited at the Department of Endocrinology of Vall d’Hebron University Hospital. However, nine patients were not included in the final analysis due to dropping out mid-study (*n* = 5) or due to not following the clinician’s guidelines requested for data acquisition (*n* = 4).

Therefore, a total of 42 patients with Type 2 diabetes were included in the study, and their data were acquired through an approved clinical trial (NCT02248311), according to the tenets of the Declaration of Helsinki. The ethic committee of the Vall d’Hebron University Hospital approved all procedures (protocol number PR(AG)01/2017); written informed consent was obtained for all the participants.

Inclusion criteria: (i) diagnosis of Type 2 diabetes from at least 5 years before the screening, (ii) patients with Type 2 diabetes controlled from at least a year before the scanning with stable glycosylated haemoglobin (around 7.5% but <8%) and plasma glucose levels in fasting conditions below 150 mg/dl and (iii) age from 50 to 79 years old.

Exclusion criteria: (i) Type 1 diabetes, (ii) any previous cardiovascular event, (iii) any contraindication or claustrophobia for the PET/CT, (iv) any concomitant pathology related to a short life expectancy (<1 year), (v) regular, high-intake alcohol drinkers and (vi) smokers who did not stop smoking at least ≤1 year prior to recruitment.

### Biochemical analysis

Blood samples were retrieved before applying the HEC procedure and were quickly analysed at the Biochemistry Core Facilities of Vall Hebron University Hospital using standard procedures. Thus, biochemical and immunological data were obtained. Patients with missing data were not considered for statistical analysis and comparisons, and only variables with at least 80% of patients with their corresponding data were included.

Biochemical data, including insulin and glucose levels, were posteriorly used for the calculation of HOMA-IR following the equation proposed by Matthews *et al.*^23^


$$\mathrm{HOMA}\text{-}\mathrm{IR}=\frac{\mathrm{Fasting}\;\mathrm{insulin}\;(\mathrm{mU}/l)\times \mathrm{Fasting}\;\mathrm{glucose}\;(\mathrm{mmol}/l)}{22.5}.$$

### [^18^F]FDG-PET imaging and HEC

For each patient, two [^18^F]FDG-PET brain scans were acquired before and after a HEC procedure (baseline and HEC, respectively), with the clamp protocol performed as previously described by our group^24,25^ and others.^26^ Both images were obtained in random order within 2 days under the following conditions: at least 8 h of fasting and 24 h without medication. The administered [^18^F]FDG dose was 1.9 MBq/kg and was provided 60 min before image acquisition. Whole-body IS (IS_HEC_) was assessed by taking the mean glucose infusion rate during the last 40 min of the HEC procedure, as proposed by Moreno-Navarrete *et al*.^26^

A brain PET at baseline was acquired 60 min after [^18^F]FDG injection for a period of 12 min. For post-HEC brain PET acquisition, [^18^F]FDG was administered after at least 1.5 h from the start of the protocol and after three consecutive constant blood glucose measurements separated by 5 min between each measurement. PET images were acquired 60 min after [^18^F]FDG administration, similar to baseline conditions.

Image acquisition was performed in a Siemens Biograph mCT 64, with post-processing image reconstruction using a 3-order Gaussian filter of 3 iterations and 21 subsets. Further information can be found in previous publications.^24,25^

### Statistical parametric mapping

Statistical parametric mapping (SPM, version 12), a voxel-based method focusing on tissue probability maps,^27^ was used to analyse pre- and post-HEC PET images of patients with Type 2 diabetes and obtain significantly altered brain clusters. Briefly, once all the image pairs were acquired, the brain area of the clamp PET was realigned to the area of the baseline PET. Subsequently, the baseline PET image was normalized to the Montreal Neurological Institute atlas following the guidelines by the International Consortium of Brain Mapping,^28^ ensuring consistency across all studies. The transformations necessary for normalizing the baseline study to the atlas were then applied to the (realigned) clamp study. Following this, each image was normalized by dividing it by its mean value, and the corresponding HEC-baseline subtraction was calculated for each patient. The images underwent Gaussian smoothing with a filter full width at half maximum of 4 mm for statistical analysis preparation. Differences between HEC and baseline PET studies were evaluated using the subtraction image via a one-sample *t*-test with both hypermetabolic and hypometabolic contrasts. Significance was determined at *P* < 0.05 (family-wise corrected level), with a minimum cluster size of 10 voxels. The effect of disease duration was also assessed by doing a regression analysis and by comparing early (<10 years of diagnosis) versus advanced (>15 years) patients. Age and gender were added as covariates.

In this study, differences between baseline and post-HEC PET images were used to evaluate metabolic changes. Hypermetabolic (baseline-HEC) and hypometabolic (HEC-baseline) clusters were characterized using SPM relating to brain IS and IR, respectively.

### Patient phenotyping

To determine groups within the studied Type 2 diabetes population, we applied *k*-means clustering via minimization of squared Euclidean distances between centroids to the data obtained through SPM. The relationships between multiple SPM clusters were assessed, and the best separation was chosen to define the brain phenotypes of patients with Type 2 diabetes. Afterwards, the division line working as a boundary between both groups was obtained according to the orthogonal line to the trajectory between both centroids that cuts through the midpoint between centroids.

### Statistical analysis

For statistical analysis and plotting, MATLAB R2020b (MathWorks, Natick, MA, USA) was used. Spearman correlation coefficients (*r*) and *P*-values (*P*) were acquired, and scatter plots were obtained for assessing linear distributions. Group comparisons were evaluated using a Mann–Whitney analysis, and box plots with standard errors were displayed. Statistical significance was considered if *P* < 0.05, with a confidence interval of 95%. A generalized linear regression was applied to classify each phenotype (binomial target) by means of a logit link function such that ϕ=logit=P/(1−P), with *P* being the probability of the patient belonging to the Label 1 phenotype, namely, *P*(*Y* = 1). Area under the curve (AUC) after applying receiver operating curves was obtained to assess classification potential.

## Results

### Phenotyping Type 2 diabetes according to the effects of insulin on brain

Multiple clusters representing significant changes in the glucose metabolism before and after applying HEC were retrieved from SPM analysis. Some brain regions were hypometabolic at baseline and increased their [^18^F]FDG uptake after HEC, whereas others showed hyperintensity at baseline but displayed a decreased [^18^F]FDG uptake after HEC instead. Depending on the behaviour, the clusters were classified as hypometabolic at baseline (hypo)/increased at HEC (HEC-baseline, insulin-sensible clusters) or hypermetabolic at baseline (hyper)/decreased at HEC (baseline-HEC, insulin-resistant clusters).

**Figure 1:**
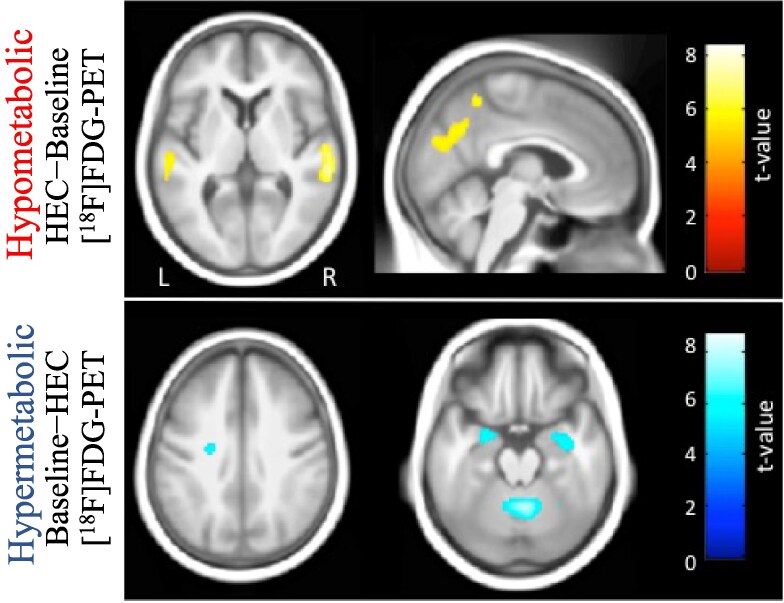
**Example of hypo- and hypermetabolic regions.** Representative sections showing the clamp effect with results overlaid over a mean structural image, the colour scale represents the corresponding *t*-value and images are displayed in neurological convention. HEC, hyperinsulinaemic euglycaemic clamp; [^18^F]FDG-PET, ^18^F-fluorodeoxyglucose positron emission tomography; L, left side; R, right side.

A total of six hypermetabolic clusters (baseline−HEC) and three hypometabolic clusters (HEC−baseline) were characterized in the brain PET images, as seen in Fig. 1 and Table 1. Figure 1 displays significant increases of [^18^F]FDG relating to glucose metabolism found at the bilateral middle temporal lobe, cuneus and precuneus. In addition, the significant decreases of [^18^F]FDG uptake after HEC were located at white matter adjacent to the left cingulum, bilateral parahippocampal gyrus and cerebellar vermis.

**Table 1:** Cluster equivalences with brain regions

Characteristic	C	Anatomical reference	Region	Function^28-30^
	1	Cerebellar vermis	Cerebellum	Locomotion and bodily posture
	2	Left parahippocampal gyrus	Limbic	Memory encoding and retrieval
Baseline-HEC	3	Right hippocampus	Limbic	Spatial memory and human learning
Hypermetabolic	4	Left amygdala	Limbic	Verbal and sustained emotional processing
Median = 0.0496	5	Left olfactory bulb	Sensory	Olfactory system, smelling information
	6	Left middle cingulate gyrus	Limbic	Processing emotions and behaviour regulation
HEC-baseline	1′	Right superior temporal gyrus	Cortex	Object- and space-related information
Hypometabolic	2′	Left superior temporal gyrus	Cortex	Language processing and auditory memory
Median = −0.1132	3′	Right precuneus	Cortex	Self-referential processing, imagery and memory

Each cluster is shown with their nomenclature, median values, anatomical reference and function of each of the studied hypermetabolic and hypometabolic clusters.

C, cluster.

**Figure 2:**
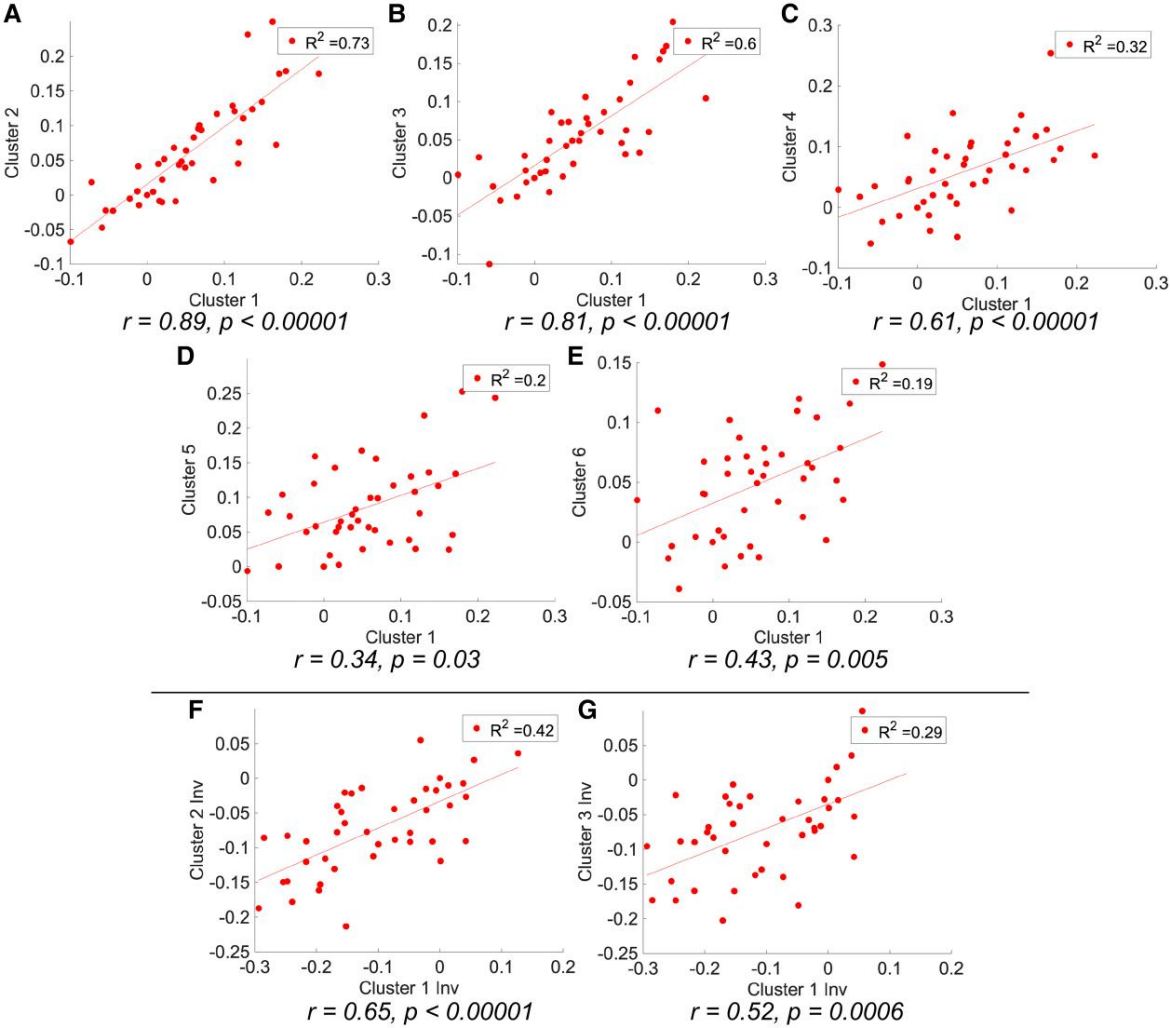
**Scatter plots representing the relationship between clusters.** SPM cluster relationships (Spearman’s correlation) between hypometabolic brain regions (clusters) with Cluster 1 as reference (**A**, **B**, **C**, **D** and **E**) and relationships between hypermetabolic brain regions (clusters) with Cluster 1′ as reference (**F** and **G**). In all cases, the number of subjects was *n* = 42.

The correlation between clusters was studied to assess their relationship. Spearman correlation analysis displayed statistically significant relationships between all clusters of the same metabolic type, with both analysed variables increasing values in a proportional manner (Fig. 2). The most significant interactions (*R*^2^ > 0.50) were observed between hypermetabolic clusters 1 and 2, 1 and 3, and 2 and 3. These correspond to the cerebellar vermis (1), left parahippocampal gyrus (2) and right hippocampus regions (3). For the case of hypometabolic clusters, the most associated (*R*^2^ > 0.40) were Clusters 1′ and 2′ and Clusters 1′ and 3′, corresponding to right (1′) and left (2′) superior temporal gyrus (STG) and the right precuneus (3′), respectively. The relationships between hypometabolic clusters and hypermetabolic clusters were not as significant (*R*^2^ < 0.30). In any case, Hypometabolic cluster 1′ and Hypermetabolic cluster 1 were the ones most significantly associated with all the other clusters.

**Figure 3:**
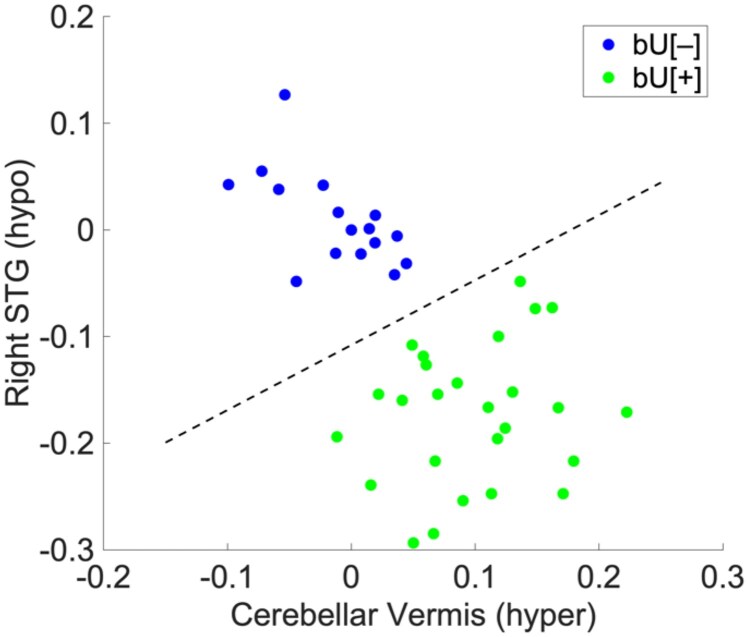
**Phenotype definition by *k*-means**. Phenotypes were best separated into two classes according to Cluster 1 and Cluster 1′, with bU[−] *n* = 16 and bU[+] *n* = 26. STG, superior temporal gyrus.

All SPM hyper- and hypometabolic clusters were compared to identify patient groups. Initially, *k*-means clustering was applied to all possible pairs of SPM clusters, including both hyper- and hypometabolic regions. Two successful classifications were obtained (Cluster 1 versus Cluster 1′ and Cluster 2 versus Cluster 1′) both yielding two matching groups. Nevertheless, Cluster 1 versus Cluster 1′ showed a clearer separation between groups. In addition, a *k*-means clustering was applied to the full set of SPM clusters, although it did not result in a successful grouping. Thus, patient brain phenotypes were defined by Hypermetabolic cluster 1 (cerebellar vermis) and Hypometabolic cluster 1′ (right STG), as seen in Fig. 3. As a result, from now on, Cluster 1 and Cluster 1′ are considered to represent the behaviour of all their metabolic-alike clusters.

Different behaviours were shown when testing cerebellar vermis and right STG values against IR systemic indices. Cerebellar vermis associated negatively with HOMA-IR (*r* = −0.31, *P* = 0.07) and positively for IS_HEC_ (*r* = 0.41, *P* = 0.009). The inverse behaviour was observed for right STG with a positive association with HOMA-IR (*r* = 0.31, *P* = 0.06) and a negative for IS_HEC_ (*r* = −0.49, *P* = 0.0012).

Differences between phenotypes were observed for HOMA-IR (*P* = 0.03) and IS_HEC_ (*P* = 0.0002), as displayed in Table 2. Phenotype I showed high HOMA-IR and low IS_HEC_ thus brain hypometabolism (bU[−]). Phenotype II associated with low HOMA-IR and high IS_HEC_, thus brain hypermetabolism (bU[+]).

**Table 2:** Patient characteristics according to brain phenotypes

Parameter	All (*n* = 42)	bU[−]	bU[+]	*P-*value
Age (years)	67 (61, 70)	66 (61, 70)	68 (60.5, 73)	0.38
Gender (M:F)	20:22	8:8	14:12	0.80
Duration (years)	12 (7, 18)	10 (7, 15)	13 (8, 19)	0.42
BMI (kg/m^2^)	30.68 (28.44, 35.3)	32.66 (30.47, 35.71)	29.55 (28.28, 33.77)	0.16
Glucose (mg/dl)	120 (110, 145)	123 (110, 152)	117.5 (110, 145)	0.66
HbA1c (mmol/l)	7.3 (6.6, 7.65)	6.95 (6.7, 7.5)	7.3 (6.45, 7.8)	0.59
RDW (fl)	13.35 (12.8, 15.5)	15.4 (13.2, 16.1)	13.2 (12.8, 13.95)	0.03
Chloride (mmol/l)	103 (102, 105.5)	103 (102, 106)	103.5 (102, 105)	0.89
Protein (g/dl)	7.05 (6.8, 7.4)	7.2 (6.9, 7.5)	6.95 (6.6, 7.4)	0.25
IL-6 (pg/ml)	2.63 (1.4, 4.54)	3.9 (1.79, 6.12)	2.02 (1.4, 3.49)	0.06
AST (U/l)	23.5 (19, 33)	29 (22, 46.5)	23 (18.5, 33)	0.07
ALT (U/l)	21 (16, 35.5)	28.5 (19, 38)	17 (15, 32)	0.04
GGT (U/l)	27 (17.5, 45)	29 (19.5, 56)	22.5 (17, 32)	0.06
HDL (mg/dl)	46.5 (38, 52)	43 (38.5, 51.5)	47.5 (38, 55)	0.28
LDL (mg/dl)	93 (80, 118)	103.5 (76.5, 128)	90 (79, 118.5)	0.62
Cholesterol (mg/dl)	164.5 (148, 200)	171.5 (145, 216)	162 (151, 189)	0.53
FFA	0.69 (0.58, 0.83)	0.69 (0.52, 0.88)	0.74 (0.58, 0.83)	0.80
TG (mg/dl)	111.5 (83, 176)	111.5 (96, 178)	112 (82, 176)	0.69
TIMP-1 (ng/ml)	259.8 (226.5, 311.2)	299.4 (230.9, 332.4)	252.1 (223.2, 299.4)	0.20
HA (ng/ml)	43.44 (30.44, 87.12)	76.41 (31.74, 94.73)	39.81 (30.33, 76.45)	0.31
PIIINP (ng/ml)	7.46 (5.72, 9.51)	8.36 (6.23, 10.93)	6.1 (5.57, 8.74)	0.049
SFRP-1 (ng/ml)	0.71 (0.37, 0.92)	0.95 (0.72, 2.16)	0.52 (0.3, 0.77)	0.007
Insulin (mU/l)	16.38 (10.53, 26.21)	19.76 (15.59, 76.21)	12.48 (9.31, 19.39)	0.04
HOMA-IR	4.98 (3.23, 9.83)	6.58 (4.74, 20.75)	4.03 (2.96, 5.45)	0.03
IS_HEC_	2.35 (1.43, 4.68)	1.44 (1.12, 2.19)	4.26 (1.82, 5.85)	0.0002

Data are indicated as median (Q1, Q3).

ALT, alanine aminotransferase; AST, aspartate aminotransferase; BMI, body mass index; FFA, free fatty acids; GGT, gamma-glutamyl aminotransferase; HA, hyaluronic acid; HbA1c, glycosylated haemoglobin; HDL and LDL, high- and low-density lipoproteins; IL-6, interleukin 6; IS_HEC_, whole-body insulin sensitivity; PIIINP, procollagen III *N*-terminal propeptide; RDW, red blood cell distribution width; SFRP-1, secreted frizzled-related protein 1; TG, triglycerides; TIMP-1, tissue metallopeptidase inhibitor 1.

**Figure 4:**
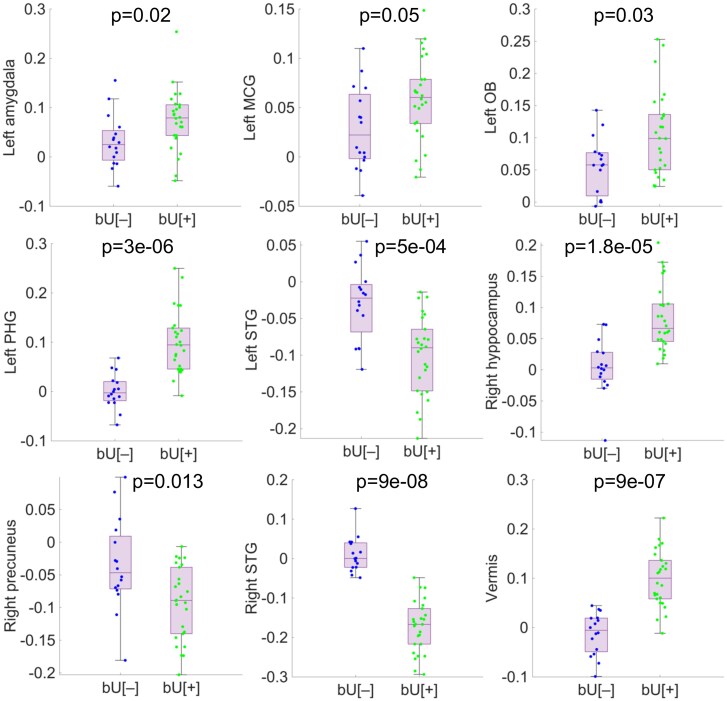
**Phenotype comparison.** Box plots displaying differences (Mann–Whitney U-test) for values of each phenotype are displayed according to clusters obtained during the SPM analysis, with bU[−] *n* = 16 and bU[+] *n* = 26. MCG, middle cingulate gyrus; OB, olfactory bulb; PHG, parahippocampal gyrus; STG, superior temporal gyrus.

Comparisons between all the SPM clusters representing hyper- and hypometabolic regions (see Table 1) were performed according to the newly defined phenotypes, which exhibited how the chosen areas represent the global brain behaviour of the six hypermetabolic and the three hypometabolic clusters. Results displayed statistically significant differences between bU[+] and bU[−] groups, with higher values in hypermetabolic regions and lower values in hypometabolic clusters for the bU[+] phenotype, as seen in Fig. 4. Thus, cerebellar vermis and right STG were considered as the representing hyper- and hypometabolic clusters, respectively, for the analysis from here on. In addition, bU[+] was associated with higher IS_HEC_ and lower HOMA-IR, thus showing lower systemic IR. On the other hand, bU[−] displayed lower IS_HEC_ and higher HOMA-IR, thus exhibiting higher systemic IR.

### Altered features between bU[−] and bU[+] phenotypes

No differences were observed between phenotypes according to gender, age or disease duration, as seen in Table 2, although multiple biochemical features were found to be different between bU[−] and bU[+]. Some were significantly different (*P* < 0.05), including red cell distribution width (RDW), alanine aminotransferase (ALT), insulin, IS_HEC_, HOMA-IR, secreted frizzled-related protein 1 (SFRP-1) and Type III procollagen peptide (PIIINP), whereas others showed slightly significant differences (0.05 < *P* < 0.10), such as interleukin 6 (IL-6), aspartate aminotransferase and gamma-glutamyl transferase (GGT).

### Predicting bU[−] and bU[+] phenotypes

**Figure 5:**
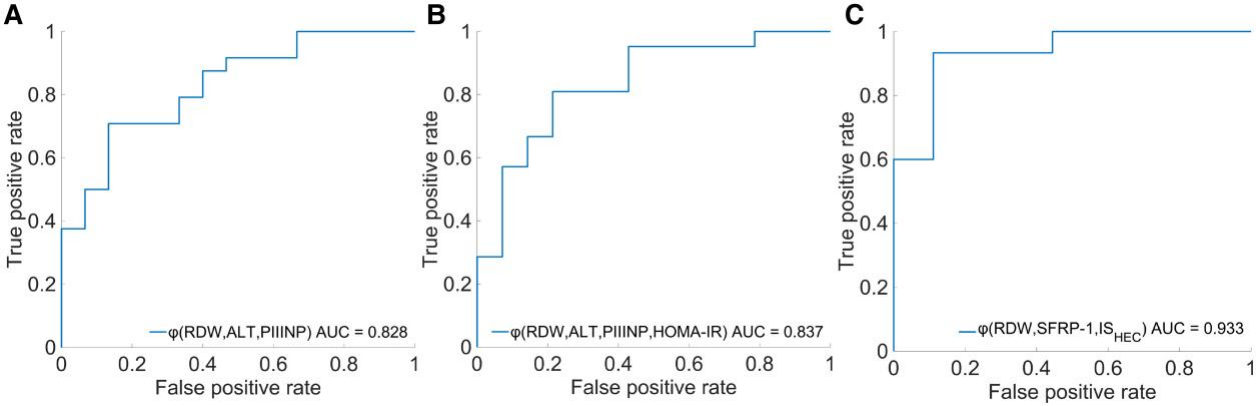
**Phenotype identification.** Classes were defined using a logit-based generalized linear model function *ϕ* built using biochemical data, including (**A**) RDW, ALT and PIIINP (*n* = 39), (**B**) RDW, ALT, PIIINP and HOMA-IR (*n* = 35) and (**C**) RDW, SFRP-1 and ISHEC (*n* = 24). The number of patients was decreased in the models as all patients had all their data available. ALT, alanine aminotransferase; HOMA-IR, homeostasis model assessment of insulin resistance; IS_HEC_, whole-body insulin sensitivity; PIIINP, Type III procollagen peptide; RDW, red cell distribution width; SFRP-1, secreted frizzled-related protein 1.

Since the [^18^F]FDG-PET HEC protocol is not available in clinical practice, and to reduce the patients’ exposure to radiation dosimetry, we developed a classification function according to bU[−] and bU[+] phenotypes, including the altered biochemical parameters, as seen in Fig. 5.

Parameters relating to Type 2 diabetes that show high intercorrelation and may be influenced between each other are only included once in the model, that is, neither the combinations of HOMA-IR and IS_HEC_ (*r* = −0.35, *P* = 0.016), ALT and SFRP-1 (*r* = −0.30, *P* = 0.03), ALT and IS_HEC_ (*r* = −0.52, *P* = 0.0002) or PIIINP and IS_HEC_ (*r* = −0.38, *P* = 0.03) were included to avoid collinearity and redundancy. Additionally, HOMA-IR depends on insulin levels, for which insulin is disregarded and only HOMA-IR is considered to avoid collinearity between terms. The included parameters have shown differences between bU[−] and bU[+] phenotypes. The outputs of a binomial classification corresponded to the probability of belonging to each phenotype and ranged between 0 and 1. Then, we have bU[+] if ≥ 0.5 (upper 50%) and bU[−] if < 0.5 (lower 50%).

The models are displayed in Fig. 5, which were built using a logit function and are displayed according to increasing complexity:

Figure 5A:


$$\begin{array}{rl} \varphi & =15.658-0.608\times \mathrm{RDW}-0.091\times \mathrm{ALT}-0.549\\ & \;\times \mathrm{PIIINP}. \end{array}$$

Results of the model with *n* = 39 have shown *P*  *=*  *0.002* and a classification performance of AUC = 0.83 [true positive rate (TPR) = 0.71, true negative rate (TNR) = 0.87].

Figure 5B:


$$\begin{array}{rl} \varphi & =13.470-0.496\times \mathrm{RDW}-0.085\times \mathrm{ALT}-0.440\\ & \;\times \mathrm{PIIINP}-0.041\times \mathrm{HOMA}\text{-}\mathrm{IR}\;. \end{array}$$

Results of the model with *n* = 35 have shown *P* = 0.009 and a classification performance of AUC = 0.84 (TPR = 0.81, TNR = 0.78).

Figure 5C:


$$\begin{array}{rl} \varphi & =12.792-0.785\times \mathrm{RDW}-2.732\times \mathrm{SFRP}1\\ & \;+0.476\times IS_{\mathrm{HEC}}. \end{array}$$

Results of the model with *n* = 24 have shown *P* = 0.0011 and a classification performance of AUC = 0.93 (TPR = 0.93, TNR = 0.89).

The best performance was observed for Model C. Nonetheless, since IS_HEC_ can only be obtained through the HEC protocol, which is not patient friendly acquired and always available, and keeping in mind that the available subjects were reduced from *n* = 42 to *n* = 24, the most useful models were considered to be A and B.

## Discussion

In the present study, we evaluated the role of insulin-mediated brain glucose metabolism in Type 2 diabetes by using two [^18^F]FDG-PET scans, at baseline (standard conditions) and after applying a HEC. Thus, we have assessed the effect of insulin on glucose uptake in different brain regions. We found, for the first time to our knowledge, several insulin-mediated glucose hypo- and hypermetabolic clusters modulated by insulin in the brain of patients with Type 2 diabetes by SPM analysis that are associated with multiple brain functions.^29-31^ For instance, the hypometabolic clusters included right STG, left STG and right precuneus, all of which are found in the cortex region. The hypermetabolic clusters included the cerebellar vermis, left parahippocampal gyrus, right hippocampus, left amygdala, left olfactory bulb and left middle cingulate gyrus, which are associated with the cerebellum, the olfactory area and the limbic region. All hypometabolic clusters were tightly related to systemic IR, whereas hypermetabolic clusters showed higher systemic IS.

Among all brain clusters affected by insulin, those that clusterized subjects into two separate phenotypes included the cerebellar vermis (hypermetabolic) and the right STG (hypometabolic). Clustering, however, depended on the relationship between both regions rather than their independent values, suggesting an interaction between them. This highlights the connections between hypometabolic and hypermetabolic regions, as previously described.^5,7-10,32,33^ However, we suggest for the first time that the cerebellar vermis and the right STG are the more relevant areas in terms of insulin-mediated glucose uptake brain affectation in Type 2 diabetes. Furthermore, these two areas were found to be representative of the behaviour of all their metabolic-alike clusters and were used for phenotyping. One phenotype displayed predominant hypometabolism whereas the other showed predominant hypermetabolism. Thus, they were considered as bU[−] and bU[+], respectively. In addition, to our knowledge, it is the first study to propose the importance of such hypo- and hypermetabolic areas and their interaction in terms of glucose metabolism for determining CD risk in Type 2 diabetes. Additionally, we suggest that transitioning from one phenotype to the other relies on the relationship between the glucose metabolism of the hypermetabolic cerebellar vermis and the hypometabolic right STG, thus defining different risk for CD.

In addition, hypermetabolic cerebellar vermis has shown a direct relationship with whole-body IS, and an inverse association with HOMA-IR, whereas the hypometabolic right STG displayed inverse and direct associations, respectively. This confirmed their relationship with IR and indicated that the cerebellum contributes less to systemic IR and becomes sensitive, whereas the right STG plays a major role in CD development due to its contribution to brain IR. This is in accordance with the observation done by Dong *et al*.^21^ where they suggested that the hypometabolism in the brain, specifically in the right middle temporal gyrus, contributes to cognitive decline and Alzheimer’s disease in a study performed between control and non-Type 2 diabetes patients with subjective cognitive decline.

It was observed how the bU[−] phenotype associated with higher systemic IR (high HOMA-IR, low IS_HEC_) whereas the bU[+] phenotype associated with lower systemic IR (low HOMA-IR, high IS_HEC_), suggesting a higher metabolic risk for the bU[−] as IR has been previously and widely associated with multiple comorbidities in Type 2 diabetes.^34^ This was predictable, as hypometabolic clusters were found to be directly associated with systemic IR indices, whereas hypermetabolic clusters were inversely related instead. However, it is highly interesting how bU phenotypes based on the relationship between hypo- and hypermetabolic clusters display a similar association. Namely, switching from bU[+] to bU[−] relies on which is the predominant area and its insulin-mediated glucose metabolism, either hyper- or hypometabolism, respectively. Thus, cerebellar vermis and right STG seem to be playing a relevant role in phenotype definition and therefore are highly important in defining an increased risk for CD.

Moreover, the cerebellar vermis is responsible for locomotion, bodily posture and sensorial functions.^30^ This area displayed glucose hypermetabolism and was predominant in bU[+]. Meadowcroft *et al*.^8^ proposed that hypermetabolism in the cerebellum associates with local neurodisruption. However, their study only considered metabolically healthy patients. Conversely, findings in this study propose that the cerebellar vermis may be influenced by sensitivity to insulin, decreasing its glucose metabolism in Type 2 diabetes, which also seems to play a role in CD. Indeed, bU[+] patients exhibited higher cerebellar vermis values, for which we suggest that hypermetabolic areas contribute to brain IS in a compensatory manner in Type 2 diabetes.

On the other hand, the right STG has been associated with memory and space-related information.^31,35^ This region exhibited glucose hypometabolism and thus was predominant in bU[−]. bU[−] subjects displayed higher right STG values when compared to bU[+], which suggests that hypometabolic areas in Type 2 diabetes could be contributing to the development of CD due to brain IR. This is in agreement with findings proposed by Dong *et al*.^21^ as glucose hypometabolism in the right middle temporal gyrus was found to correlate with Alzheimer’s disease progression in non-Type 2 diabetes patients. In addition, previous studies have also discussed the connection between Alzheimer’s disease and the hypometabolism in the middle and inferior temporal gyrus, the parahippocampal gyrus, the posterior cingulate gyrus and the parietal lobe, although the patterns seem to vary depending on what function is affected within Alzheimer’s disease.^36^ Nonetheless, our results seem to be in agreement with hypometabolism in the temporal gyrus, which would relate Type 2 diabetes with potential CD such as Alzheimer’s disease in the bU[−] phenotype where hypometabolism is predominant. Thus, these findings also strengthen the choice of the right STG as one of the phenotyping clusters that is used to separate into higher risk for CD.

Furthermore, each newly defined phenotype correlated differently with systemic IS. For instance, bU[−] patients exhibited lower insulin-mediated glucose uptake related to increased whole-body IR. Nonetheless, bU[+] subjects related to both increased insulin-mediated glucose uptake related to whole-body IS instead. This suggests that the brain is an IS organ that contributes to body glucose homeostasis at both organ and systemic levels, as expected.^11^ In addition, bU[−] patients might be more exposed to risk of CD as brain IR has been linked to CD in the past, including Alzheimer’s disease.^13^

All CD assumptions come from the alteration of SFRP-1, as elevated levels of this protein have been found to be directly associated with Alzheimer’s disease and other CD.^37^ The bU[−] phenotype displayed elevated SFRP-1,^37-39^ suggesting that this phenotype has more risk of CD than the bU[+]. On the other hand, features related to Type 2 diabetes physiopathology, including HOMA-IR, IS_HEC_ and plasma concentration of insulin^23,40-43^ were altered as well, suggesting that these patients have more severe Type 2 diabetes. In addition, ALT values were also altered with higher values for bU[−], which has been associated with Type 2 diabetes and IR in previous publications.^44^ Nonetheless, ALT values have been shown to be decreased for patients with Alzheimer’s disease and CD.^45^ Thus, it appears that Type 2 diabetes with potential CD may display different biomarker patterns than CD alone. Regarding the specific features of each phenotype, altered biochemical parameters were observed for bU[−] subjects, which related to systemic IR (increased insulin and HOMA-IR and decreased IS_HEC_^41^), neuroinflammation and Alzheimer’s disease (increased RDW^46^ and SFRP-1^37,39,47^) and memory dysfunction (tranferases such as elevated ALT and GGT levels^45,48^). Altogether suggests that bU[−] Type 2 diabetes phenotype associates with systemic markers of neuroinflammation, related to enhanced risk of CD. As a result, decreased post-HEC [^18^F]FDG uptake relating to decreased glucose metabolism and thus brain IR associates with cognitive impairment, which is supported by previous findings relating hypoenhanced regions of the brain with CD, including Alzheimer’s disease.^7^

The altered biochemical parameters between both phenotypes enabled the construction of a classification model aiming at detection of bU[−] patients. Thus, a classification function relied on biochemical data retrieved from blood tests was obtained and displayed a high accuracy for its potential use in clinical practice after its validation in large cohorts. This may allow the development of individualized CD risk prevention strategies in Type 2 diabetes in the future, thus contributing to personalized medicine for this specific type of patients. We highlight the importance of this since Type 2 diabetes is a risk factor for CD pathologies causing devastating mental health issues for patients and an increased cost in the healthcare system.

### Strengths and limitations

Included patients belonged to a clinical trial that guaranteed randomized and controlled patient selection, and the present study used HEC-[^18^F]FDG-PET to measure *in vivo* brain-specific IR to evaluate specific hypo- and hypermetabolic regions that could relate to CD in Type 2 diabetes. Nonetheless, certain limitations of the study need to be taken into consideration: (i) the classification models relied on PIIINP, which is not available from simple serological tests, (ii) subject selection was limited to 50–79 year olds and did not include control subjects nor consider prospective data reflecting the evolution of the patients and (iii) it did not consider ethnicity, socio-economic status, medication or lifestyle, which limits the validity of the model to the studied population. Validation with an external cohort would increase its reliability. In addition, partial volume effect correction was not applied due to the unavailability of magnetic resonance, which is typically required to accurately delineate brain structures for correction. While PET-only methods exist for partial volume effect correction, they have been observed to introduce noise and uncertainties to the images, as highlighted by Erlandsson.^49^ Thus, in the context of this study, such corrections could reduce the reliability of the results.

## Conclusions

In summary, we confirmed the existence of hypo- and hypermetabolic glucose patterns in brain regions of patients with Type 2 diabetes. This allows for successfully phenotype patients with Type 2 diabetes into bU[−] or bU[+] using brain [^18^F]FDG uptake of predominant hypometabolic (right STG) or hypermetabolic (cerebellar vermis) regions, respectively. Thus, switching from one bU phenotype to the other relies mainly on the insulin-mediated glucose metabolism in these two interacting areas. Altered parameters between both phenotypes enabled the building of a non-invasive phenotyping model to detect bU[−] and bU[+] subjects based on patient-friendly and accessible biochemical parameters. As a result, its implementation into clinical practice could contribute to future personalized treatment strategies and CD risk prevention in Type 2 diabetes.

## Data Availability

The data included in this study can be made available from the corresponding author upon reasonable request.
